# Revealing Electrical and Mechanical Performances of Highly Oriented Electrospun Conductive Nanofibers of Biopolymers with Tunable Diameter

**DOI:** 10.3390/ijms221910295

**Published:** 2021-09-24

**Authors:** Muhammad A. Munawar, Dirk W. Schubert

**Affiliations:** 1 Institute of Polymer Materials, Department of Material Science, Faculty of Engineering, Friedrich-Alexander-University Erlangen-Nuremberg, Martensstrasse 7, 91058 Erlangen, Germany; 2 KeyLab Advanced Fiber Technology, Bavarian Polymer Institute, Dr.-Mack-Strasse 77, 90762 Fürth, Germany

**Keywords:** Young’s modulus, electrical percolation threshold, fiber diameter, orientation, biomedical

## Abstract

The present study outlines a reliable approach to determining the electrical conductivity and elasticity of highly oriented electrospun conductive nanofibers of biopolymers. The highly oriented conductive fibers are fabricated by blending a high molar mass polyethylene oxide (PEO), polycaprolactone (PCL), and polylactic acid (PLA) with polyaniline (PANi) filler. The filler-matrix interaction and molar mass (*M*) of host polymer are among governing factors for variable fiber diameter. The conductivity as a function of filler fraction (*φ*) is shown and described using a McLachlan equation to reveal the electrical percolation thresholds (*φ_c_*) of the nanofibers. The molar mass of biopolymer, storage time, and annealing temperature are significant factors for *φ_c_*. The Young’s modulus (*E*) of conductive fibers is dependent on filler fraction, molar mass, and post-annealing process. The combination of high orientation, tunable diameter, tunable conductivity, tunable elasticity, and biodegradability makes the presented nanofibers superior to the fibers described in previous literature and highly desirable for various biomedical and technical applications.

## 1. Introduction

The orientation, diameter, electrical conductivity, and mechanical strength (elasticity/stiffness) are among the most significant characteristics of electrospun fibers for reliable usage in various biomedical applications: cardiac tissue [1,2,3,4,5,6,7], muscle tissue [8,9,10], nerve tissue [11,12,13,14,15], bone tissue [16,17], wound healing [18,19], etc., and technical applications and wearable electrical devices [20,21], brain-machine interface [22], biomedical devices [23,24], field-effect transistors (FET) [25], biosensors [26,27,28] and electrodes [29,30,31]. In recent years, highly aligned nanofiber arrays have become some of the most prominent nanostructures owing to their directionality [32]. Moreover, the highly aligned fibers can provide directional guidance during the release and growth of proteins, especially in tissue engineering fields, such as neurite and muscle outgrowth [33].

Fiber diameter has a significant effect on the differentiation and proliferation of cells. Christopherson et al. [34] suggested that rat neural stem cells (rNSCs) preferentially differentiate into oligodendrocytes on smaller fiber-diameter substrate, while on larger fiber-diameter substrate, rNSCs preferentially differentiate into neuronal lineage. This allows investigators to tailor fiber diameter according to the desired cell response.

Electrical admittivity of human tissue may be predominantly conductive, capacitive, or a combination of these depending on tissue type, availability of charge carriers, and frequency of the applied electric field. It is carried out by ions, dipoles, or electrons as well as holes (semiconductor) [35,36,37]. As such, electrical activity is a key feature of many types of tissues and organs, such as skin, heart, muscle, nerve, bone, cartilage, and cornea [38]. The conductivity can be enhanced by increasing ionic strength or through the addition of electroconductive materials, including nanoparticles, nanorods, nanowires, carbon nanotubes, and intrinsically conductive polymers (ICPs) [3,39,40,41]. The impact of electrical inducement on tissues has been known since the 1960s, when Bassett et al. proved that electrical stimulation affects bone formation [42]. It has been demonstrated that in-vitro application of electrophysiologically DC fields (1–10 V/cm) and AC currents (10 to 100 mA) governs cellular behavior via interference in migration, cytoskeleton organization, alignment of neural cells, vascular endothelial, cardiofibroblasts, and myoblast cells and enhances neurite outgrowth in nerve cells, differentiation, and collagen production [43]. Preliminary studies have confirmed that the electrical properties of implanted scaffolds must be tuned correctly for the development of physiologically suitable artificial tissues.

The electrospun scaffold provides a “house” for cells and can regulate cellular function and behavior via cell-scaffold interactions. The mechanical cues that cells experience are intimately related to the microstructure of the scaffold. A well-known example is that stem cells differentiate into specific lineages (from neurogenic to osteogenic) depending on the relevant mechanical properties of the scaffold (from brain to collagenous bone) [44]. Additionally, physical, biochemical, and mechanical stimulations affect cell-material interactions potentially to guarantee the required regeneration [45]. Tissue properties, such as stiffness and biosignals, determine cellular activity, including adhesion, proliferation, differentiation, and growth. Thus, a complete understanding of mechanobiology is crucial to the appropriate use of electrospun fibers in biomedical applications.

The most studied ICPs in the biomedical field are PANi and polypyrrole (PPy). The dispersibility of PANi and PPy in water is limited, and therefore, their potential use is also limited [6]. Polythiophene family members are also an attractive alternative for various biomedical applications, as they exhibit improved dispersibility in water [46]. The common attributes of ICPs, such as electroactivity, reversible oxidation, low density, adjustable electrical conductivity (10^−11^ to 10^5^ S cm^−1^), hydrophobicity, biocompatibility, and surface topography [3,39,40,41], are required for tissue engineering and regenerative medicine applications. However, the combination of conductivity, electroactivity, biostability, and biocompatibility of ICPs blended with biopolymers (polyethylene oxide PEO, polycaprolactone PCL, polylactic acid PLA) provides an appropriate platform for various electrically stimulated biosensing applications [47]. One-dimensional (1D) materials produced by electrospinning are superior to conventional film-based planar materials having large specific surface area and suitable mechanical Young’s modulus (stiffness) [48,49].

Moreover, the ICPs can form a percolating pathway for electrons and encourage the formation of electron-hole pairs, which can improve the electronic performance of electrospun conductive fiber composites [50].

The electrical percolation threshold is the critical volume fraction (*φ_c_*) of conductive filler in electrospun conductive fiber composites (ECFCs) at which the electrical conductivity increases by several orders of magnitude. Below this critical volume fraction, the ECFCs cannot form a continuous path, meaning the conductivity of the material is extremely low. However, conductivity increases above *φ_c_* and gradually tends to a constant value. This is the conductivity percolation phenomenon. It is important to determine this conductivity percolation because the materials exhibit an insulator–conductor transition; specifically, the electrical conductivity rapidly increases when initial conductive channels are formed. A detailed study was conducted for determining the electrical percolation threshold of highly oriented electrospun conductive fibers in our recent article [51]. The percolation phenomenon depends on filler–matrix interactions, molar mass of matrix, inherent electrical conductivity of the filler, and post-annealing conditions.

In this study, the highly oriented electrospun conductive fiber composites (ECFCs) of PEO, PCL, and PLA blended with doped PANi were fabricated using an electrospinning process. The electrospinning set-up deploying a proprietary designed rotating wheel collector enabled high orientation of ECFCs [52]. The highly oriented ECFCs were collected on microscopic glass slides (GS) for density, resistance (conductivity), and elasticity measurements. A new procedure and model equations were developed for the calculation of conductivity (*σ*) and Young’s modulus (*E*) of ECFCs. The values for electrical percolation threshold *φ_c_* were determined by fitting the McLachlan general effective media (GEM) equation to experimental conductivities for all types of fabricated ECFCs. The slope of the force-strain curve of a bundle of conductive fibers was determined in the linear elastic region and used for calculating the Young’s modulus. The Young’s moduli as a function of filler fraction were determined using a density-based model equation. A subsequent annealing was conducted, varying annealing temperature and time to show effects on percolation thresholds and Young’s moduli of ECFCs, respectively.

The main motivation of this study was to determine the electrical and mechanical properties of conductive fibers using the new, simple, and reliable methods considering fiber dimension and alignment.

## 2. Results and Discussion

### 2.1. High Orientation with Tunable Fiber Diameter

**Figure 1:**
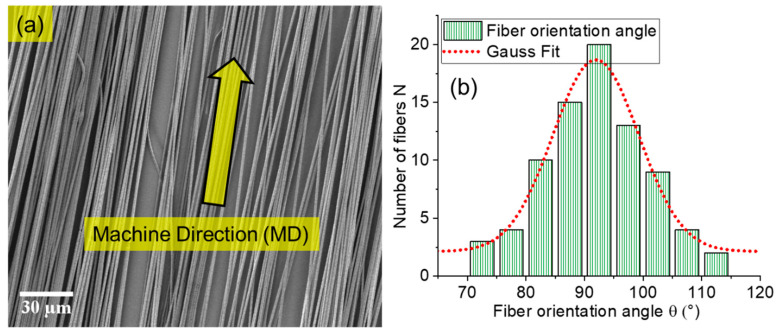
Panel (**a**) shows SEM image with high orientation of electrospun fibers. Panel (**b**) shows directionality analysis with the histogram and normal distribution curve (Gauss Fit) of orientation angle. The class width is approximately 5°. 90° is positioned in the middle of the class because the fibers were aligned to the machine direction MD.

The highly aligned electrospun conductive nanofibers of biodegradable polymers were fabricated successfully using a special collector electrode. The scanning electron microscopy (SEM) image (Figure 1a) shows that the fibers are straight and highly aligned in the machine direction MD, while the orientation angles of the fibers were measured against the vertical direction in the SEM image because the vertical axis of the image was positioned to coincide with the machine direction. The histogram (Figure 1b) shows that the fibers are predominantly oriented between 70° and 115°. The highest number of fibers was collected at an angle of 90°, and the orientation angle distribution can be described by a normal (Gauss Fit) distribution.

**Figure 2:**
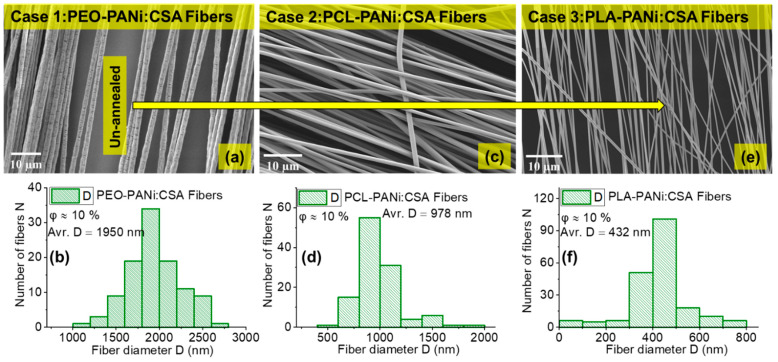
SEM images with their fiber-diameter histograms for Case 1: PEO-PANi:CSA fibers (panels **a**,**b**), Case 2: PCL-PANi:CSA fibers (panels **c**,**d**), and Case 3: PLA-PANi:CSA fibers (panels **e**,**f**), respectively. The concentration of filler PANi (*φ_PANi_* ≈ 10%) is the same for all cases, and all fibers are un-annealed.

**Figure 3:**
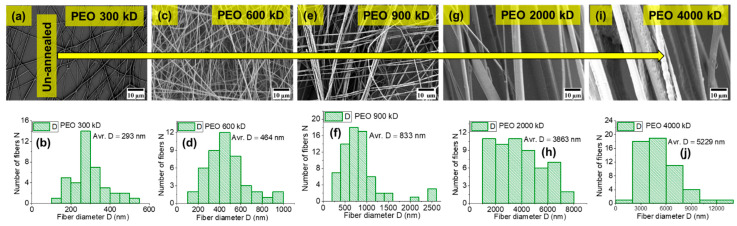
The Panels (**a**–**j**) represent the SEM analysis for PEO-300 kD, PEO-600 kD, PEO-900 kD, PEO-2000 kD, and PEO-4000 kD fibers with their fiber-diameter histograms, respectively. The concentration of PEO matrix is fixed up to 2% (*φ_PEO_* ≈ 2%) in spinning solutions for all molar masses (300–4000 kD). Note: A small misalignment of fibers is an issue when handling fibers during preparation of samples for SEM measurements.

A detailed SEM image and statistical analysis was conducted for PEO-PANi:CSA fibers (Figure 2 and Figure 3), PCL-PANi:CSA fibers (Figure 2) and PLA-PANi:CSA fibers (Figure 2), respectively. Figure 2 and Figure 3 show the SEM images with their fiber-diameter histograms, including filler concentration (*φ*%) and average fiber diameter (avr. *D*) for Case 1–Case 3. Note that the fiber diameter varies from case to case. In this study, the filler–matrix interaction and molar mass (*M*) of matrix are significant factors that affect the fiber diameter (*D*) of conductive fibers independently. The effect of filler–matrix interaction on fiber diameter is apparent from Figure 2, where the filler (PANi) produces variable fiber diameter with PEO, PCL, and PLA matrices (50 nm < *D* < 2500 nm) under the same processing conditions. However, filler-matrix interaction and filler concentration ratio are responsible for the variable diameter and have stated in our recent study [51]. 

Moreover, the order of molar mass *M* of polymer matrix is PEO > PCL > PLA and the corresponding trend in fiber diameter is observed: 1950 nm > 978 nm > 432 nm for cases 1, 2, and 3, respectively. Figure 3 shows the trend in fiber diameter for PEO fibers for different molar masses of PEO (300 kD, 600 kD, 900 kD, 2000 kD, and 4000 kD), with *φ_PEO_* ≈ 2%. The fiber diameter (*D*) increases (from 290 nm to 5200 nm) with increasing molar mass of PEO. Higher molar mass provides higher viscosity of the polymer solution, which is responsible for thicker fiber diameter when using electrospinning. The statistical analysis (Figure 2 and Figure 3) show that at least bimodal fiber-diameter distribution is present as a result of jet splitting during the electrospinning process. The dependency of viscosity and concentration on the fiber diameter and jet splitting for bimodal distribution has been discussed theoretically and experimentally in [53,54], respectively. However, the molar mass is also among significant factors for determining fiber diameter. The fiber-diameter (*D*) range of our highly oriented conductive fibers is approximately 50 nm < D < 5000 nm, making them desirable for various applications.

### 2.2. Identifying the Electrical Percolation Thresholds

The general effective media (GEM) equations (Equations (1) and (2) presented by McLachlan) [55] are used to determine the critical volume fraction/percolation threshold (*φ_c_*) of ECFCs:

(1) $$\left(1-\varphi \right)\cdot \frac{\sigma_{m}^{1/s}-\sigma^{1/s}}{\sigma_{m}^{1/s}+\left(\frac{1-\varphi_{c}}{\varphi_{c}}\right)\cdot \sigma^{1/s}}\;+\;\varphi \cdot \frac{\sigma_{f}^{1/t}-\sigma^{1/t}}{\sigma_{f}^{1/t}+\left(\frac{1-\varphi_{c}}{\varphi_{c}}\right)\cdot \sigma^{1/t}}=0$$

(2) $$\begin{array}{c} \left(100-\varphi \right)\cdot A\;+\;\varphi \cdot B=0\;\\ \mathrm{where}\;A=\frac{\sigma_{m}^{1/s}-\sigma^{1/s}}{\sigma_{m}^{1/s}+\left(\frac{100-\varphi_{c}}{\varphi_{c}}\right)\cdot \sigma^{1/s}}\;\mathrm{and}\;B=\frac{\sigma_{f}^{1/t}-\sigma^{1/t}}{\sigma_{f}^{1/t}+\left(\frac{100-\varphi_{c}}{\varphi_{c}}\right)\cdot \sigma^{1/t}} \end{array}$$

where *φ* is the volume fraction of filler in solid ECFCs calculated using Equation (S10) (derived and stated under Supplementary materials in Section S1), *σ* is the conductivity of ECFCs, *σ_m_* and *σ_f_* are the conductivities of pure matrix and filler, respectively (stated under Table 1), and *φ_c_* characterizes the percolation threshold. *s* and *t* are universal constants [56].

To convert volume fraction (*φ*) to volume percent (*φ*%) of filler, Equation (1) can be re-written as Equation (2);

Graphs are plotted utilizing logarithm of conductivity (*log* *σ*) of ECFCs and volume percent of filler (*φ*%). The GEM equation (Equation (2)), with its universal exponents *s* = 0.87 and *t* = 2 [51], is fitted with adjustable *φ_c_*, *σ_m_*, and *σ_f_* on each set of experimental conductivities of ECFCs in all three cases. Nevertheless, the fit is performed under the constraints that the conductivities of matrix (*σ_m_*) and filler (*σ_f_*) must have constant value within each case. Thus, in the McLachlan GEM equation-fitting procedure, *φ_c_* is the only adjustable parameter, while the other parameters (*s*, *t*, *σ_m_*, and *σ_f_*) are kept constant.

To check the annealing dependence of the conductivity of ECFCs, PEO-PANi:CSA fibers were annealed at 50, 60, 65, 70, and 75 °C for a fixed time interval of 24 h under vacuum conditions. The annealing causes lowering the percolation threshold with significant increase in conductivity of electrospun fibers.

The details of the annealing process are stated under Supplementary materials in Section S2.

The resistance *R* of annealed ECFCs was also measured at room temperature. To ensure accuracy and precision, each sample of ECFCs was fabricated, annealed, and measured at least three times.

Three cases were investigated, and all showed a steep increase in electrical conductivity on reaching a critical concentration, which corresponds to *φ_c_.* Moreover, the GEM equation was fitted only when electrical conductivity reached flat-plateaus (almost flat-plateaus) at sufficiently high degree of filler. The three cases are Case 1: PEO-PANi:CSA fibers, Case 2: PCL-PANi:CSA fibers, and Case 3: PLA-PANi:CSA fibers.

In all three cases, PANi was used as filler with equal weight doping ratio with CSA (PANi: CSA = 1:1), with conductivity of *σ_f_* = 100 ± 5 S cm^−1^. The PEO, PCL, and PLA are used as matrix in Case 1, Case 2, and Case 3, respectively. The conductivity of pure matrix (*σ_m_*) for PEO, PCL, and PLA fibers is determined at room temperature; *σ_PEO_* = (8.14 ± 0.3) · 10^−8^ *S cm^−1^*, *σ_PCL_* = (9.62 ± 1.4) · 10^−8^ *S cm^−1^*, and *σ_m_* = (1.39 ± 0.95) · 10^−^*^7^ S cm^−1^*, respectively.

**Figure 4:**
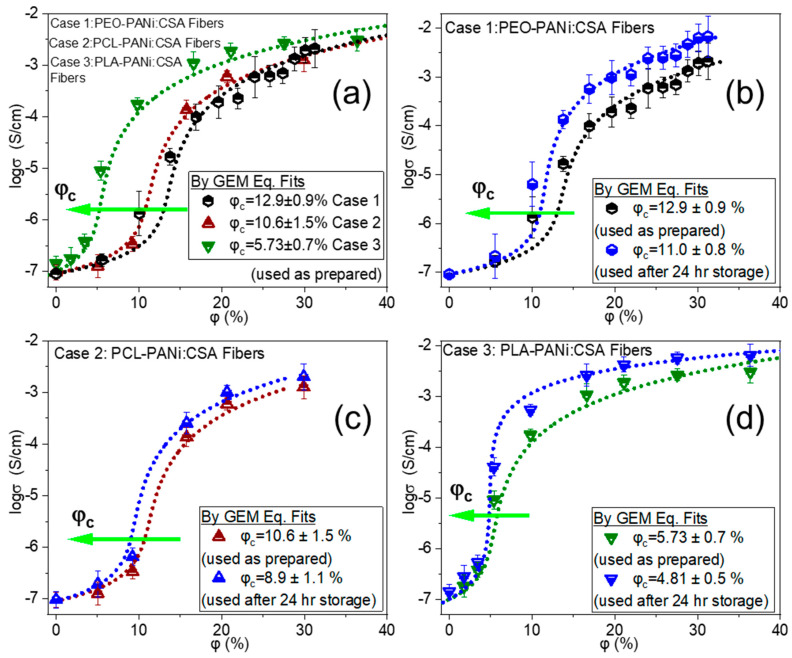
Logarithm of conductivity (*log σ*) against percentage volume fraction (*φ*%) of PANi for PEO(900kD)-PANi:CSA fibers (Case 1), PCL-PANi:CSA fibers (Case 2), and PLA-PANi:CSA fibers (Case 3), respectively. Panel (**a**) shows the comparison between all three cases (Case 1–Case 3). Panels (**b**–**d**) show the storage-time dependence for Case 1, Case 2, and Case 3, respectively. The samples are stored at room temperature for 24 h. The dotted lines show the closest fits to the GEM model (Equation (2)).

The maximum concentrations of doped-PANi, spun in Case 1, Case 2, and Case 3 is approximately 31%, 30%, and 36%, respectively. The GEM fitted curves and values of *φ_c_* for Case 1–Case 3 are shown in Figure 4. The green arrows in Figure 4 indicate the trend of decreasing *φ_c_*.

**Figure 5:**
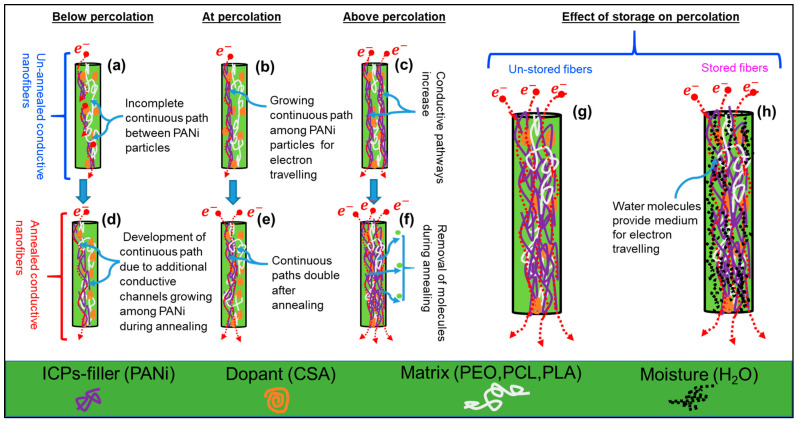
Schematic representation of percolation phenomenon within electrospun conductive nanofibers with and without annealing. Panel (**a**) elaborates the incomplete continuous path between PANi particles (below the critical concentration/percolation threshold). Panel (**b**) shows the completion of continuous path between ICPs particles for percolation phenomenon (at percolation threshold). Panel (**c**) shows that continuous pathways increase with increasing filler concentration. Panel (**d**) shows the development of continuous path due to generation of additional conductive channels among PANi particles during annealing. Panel (**e**) shows continuous paths doubling after annealing. Panel (**f**) shows continuous paths doubling due to physical/chemical interactions among PANi, CSA and PEO/PCL/PLA species with or without the removal of molecules at higher annealing temperature (above 100 °C). Panels (**g**,**h**) show the freshly prepared (un-stored) and stored (storage at room temperature for 24 h) conductive fibers. In stored conductive fibers, the water molecules provide a medium between PANi particles for electron travelling.

The variation of electrical conductivity of ECFCs with increasing filler (ICPs) concentration was divided into three stages. In the first stage, using very low concentration ICPs, the electrical conductivity of ECFCs was very low due to the non-completion of a conductive path between ICP particles (as schematically shown in Figure 5a). In the second stage, the electrical conductivity of ECFCs increased markedly, as the complete electrically conductive path formed due to mutual contacts between ICPs (schematic representation is shown in Figure 5b). The volume fraction of ICPs at this stage is called the percolation threshold *φ_c_* and calculated by fitting the McLachlan Equation. In the final stage, with the further addition of ICPs to the polymer matrix, several electrically conductive paths were formed (as schematically shown in Figure 5c), and the electrical conductivity of ECFCs further increased gradually, until levelling off.

#### 2.2.1. Significant Factors in Determining Percolation Thresholds

The various characteristics of filler and matrix, such as interactions/compatibilities between filler and host polymer and molar mass of the host polymer, have significant influence on determining percolation threshold of electrospun conductive nanofibers.

Figure 4a shows the comparison between all three cases (Case 1–Case 3) of un-annealed (used as prepared) ECFCs with their GEM equation-fitted curves. All three cases have different values of percolation threshold *φ_c_*. This is a direct consequence of different interactions/compatibilities between filler PANi and polymeric matrices of PEO, PCL, and PLA and we have already stated in our recent study [51].

**Figure 6:**
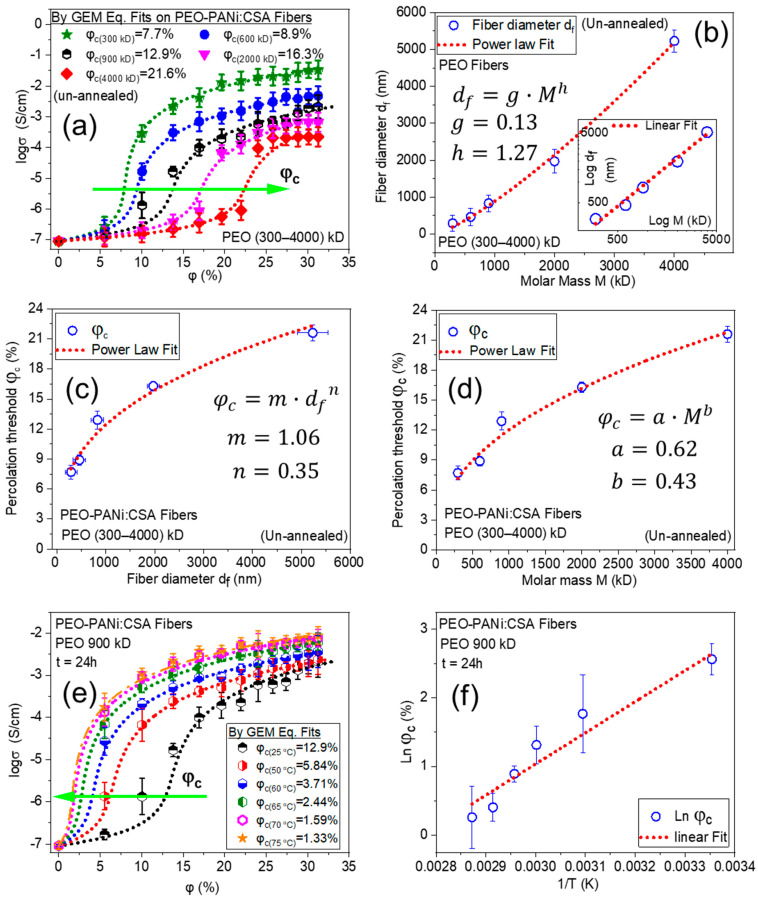
Logarithm of conductivity (*log σ*) against percentage volume fraction (*φ*%) of PANi for PEO-PANi:CSA fibers. Panel (**a**) shows the trend in *φ_c_* for PEO-PANi:CSA fibers for different molar masses of PEO (300 kD, 600 kD, 900 kD, 2000 kD, and 4000 kD). Panel (**b**) shows the power law correlation between molar mass (*M*) and fiber diameter (*d_f_*) with a coefficient g = 0.13 and exponent h = 1.27. The inset graph shows the linear fit of Log d_f_ − Log M. Panel (**c)** shows a power law correlation between fiber diameter (*d_f_*) and percolation threshold (*φ_c_*) with a coefficient m = 1.06 and exponent *n* = 0.35. Panel (**d**) shows a power law dependence of percolation threshold *φ_c_* on molar mass *M* for PEO-PANi:CSA fibers with coefficient a = 0.62 and exponent b = 0.43. Panel (**e**) shows the temperature *T* dependence on percolation threshold *φ_c_* of PEO(900 kD)-PANi:CSA fibers. The annealing is conducted at 50, 60, 65, 70, and 75 °C for 24 h. The linear relation between natural logarithm of percolation threshold (*Ln φ_c_*%) and inverse of the annealing temperature (1/T in Kelvin) indicates an Arrhenius activation process (**f**).

The order of molar mass *M* of host polymers in these three cases is PEO > PCL > PLA, and the corresponding trend in *φ_c_* values is observed; 12.9% > 10.6% > 5.73% for Case 1, 2, and 3, respectively (as shown in Figure 4a). In order to go deeper into the analysis of molar mass dependence with percolation threshold, PEO-PANi:CSA fibers were produced for different molar masses of PEO. Figure 6a shows the trend in *φ_c_* for PEO-PANi:CSA fibers for different molar masses of PEO (300 kD, 600 kD, 900 kD, 2000 kD, and 4000 kD). *φ_c_* increases with increasing molar mass of PEO. The green arrow in Figure 6a indicates the trend of increasing *φ_c_* (from 7.7 to 21.6%). Higher molar mass leads higher viscosity, which is responsible for higher incompatibility among fillers and matrices. Moreover, higher molar mass of host polymer leads to thicker fiber diameter using electrospinning and the power law correlation between molar mass and fiber diameter is shown in Figure 6b. The fibers with thicker diameters need higher percolation thresholds for developing continuous pathways for charge transport and the power law correlation between fiber diamter and percolation threshold is shown in Figure 6c. However, the direct correlation between molar mass (*M*) and percolation threshold *φ_c_* is shown in Figure 6d. The percolation threshold *φ_c_* has a power law dependency with molar mass *M*. 

#### 2.2.2. Post Factors for Determining Percolation Thresholds

Post factors, such as storage time (stored at room temperature 15–25 °C and relative humidity 20–70%) and annealing temperature also have significant influence on determining percolation thresholds of ECFCs. Due to the high specific surface area of electrospun fibers and high affinity of polyaniline for water molecules, high inter-grain connectivity is possible, which ultimately increases electrical mobility in stored ECFCs (schematic representation is shown in Figure 5h). Figure 4b–d show that storage has a marginal effect on percolation threshold *φ_c_* of ECFCs; *φ_c_* decreases with storage time. Moreover, for Case 1, the storage effect is more pronounced (*φ_c_* decreases from 12.9 to 11%) because PEO is more hydrophilic than PCL and PLA.

During the annealing process, the molecular chains of host polymers become more relaxed (expanded), and ICPs (PANi particles) acquire a certain degree of freedom to interconnect/reorganize themselves. This reorganizing (intermingling) of ICPs generates more paths and additional conductive channels (as schematically shown in Figure 5d–f), which are responsible for inter and intra-chain conductivity in annealed ECFCs [57,58]. Nevertheless, annealed ECFCs can transport charge more efficiently than un-annealed. Therefore, the annealed ECFCs have higher conductivity with lower percolation threshold *φ_c_* than un-annealed ECFCs [59,60,61,62]. The effect of annealing temperature on PEO (900 kD)-PANi:CSA fibers is shown in Figure 6e. The green arrow in Figure 6e indicates the trend of decreasing *φ_c_* (from 12.9 to 1.3%). The correlation between percolation threshold *φ_c_* and temperature (*T*) is shown in Figure 6f indicating an Arrhenius activation process within experimental error.

### 2.3. Identifying the Young’s Modulus

**Figure 7:**
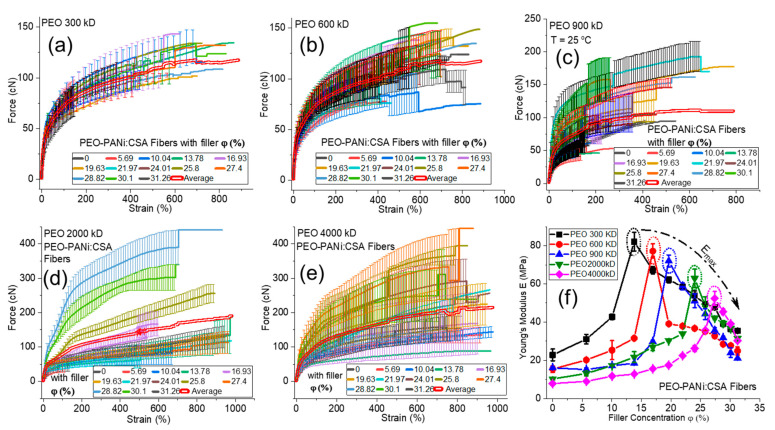
Panels (**a**–**e**) show the force-strain curves for PEO-PANi:CSA fibers using 300 kD–4000 kD molar mass of PEO. The percentage volume fraction (*φ*%) of PANi varies from 0–31% for each molar mass of PEO. The bold double red lines (**a**–**e**) represent the average of all force-strain curves. Panel (**f**) shows elastic Young’s modulus (*E*) as a function of percentage volume fraction (*φ*%) of PANi for PEO-PANi:CSA fibers at each molar mass of PEO (300 kD–4000 kD). The dotted circles (**f**) represent the maximum elastic Young’s modulus (*E_max_*) for each molar mass.

Figure 7a–e show the force-strain curves for PEO-PANi:CSA fibers using 300 kD–4000 kD molar mass of PEO for 0–31 *φ*% (percent volume fraction) of PANi, respectively. Figure 7f shows the trending curves of Young’s modulus/elasticity (*E*) for different molar mass of PEO as a function of *φ*% of PANi. The elasticity *E* of PEO-PANi:CSA fibers increases to maximum Young’s modulus *E_max_* (as shown within the dotted circles in Figure 7f) and then decreases as a function of *φ*% of PANi for each molar mass of PEO. The decreases in mechanical strength after the optimal concentration of PANi are expected due to the consequences of phase separation between polymeric matrix PEO and filler PANi particles.

**Figure 8:**
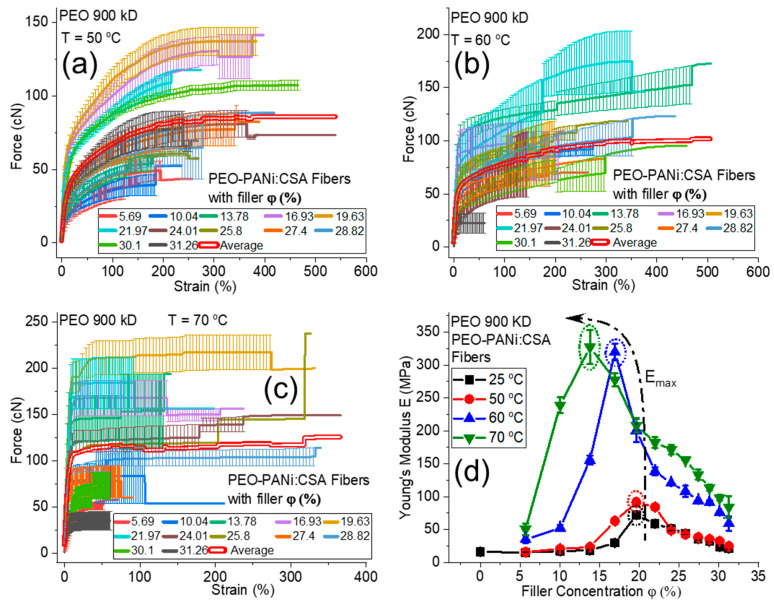
Panels (**a**–**c**) show the force-strain curves for PEO (900 kD)-PANi:CSA fibers using 50, 60, and 70 °C annealing temperatures, respectively. The percentage volume fraction (*φ*%) of PANi varies from 0–31%. The bold double red lines (**a**–**c**) represent the average of all force-strain curves. Panel (**d**) shows elastic Young’s modulus (*E*) as a function of percentage volume fraction (*φ*%) of PANi for PEO(900 kD)-PANi:CSA fibers at 25 (un-annealed), 50, 60, and 70 °C annealing temperature. The dotted circles (**d**) represent the maximum elastic Young’s modulus (*E_max_*) for each annealing temperature.

Figure 8a–c show the force-strain curves for PEO (900 kD)-PANi:CSA fibers using 50–70 °C annealing temperature for 0–31 *φ*% of PANi, respectively. Figure 8d shows the trending curves of *E* for un-annealed (25 °C) fibers and for 50, 60, and 70 °C annealing temperatures as a function of *φ*% of PANi, respectively. On annealing, *E* also increases to *E_max_* (as shown within the dotted circles in Figure 8d), then decreases as a function of *φ*% of PANi for each annealing temperature. The annealing after optimal concentration of filler particles is also responsible for the initiation of crack growth behavior, which leads to lower mechanical strength of conductive fibers.

#### Factors Affecting Young’s Modulus

The mechanical properties of fibers (especially elasticity) are influenced by many factors, but filler concentration, molar mass, and post annealing are the most significant in the present study. The Young’s modulus *E* of PEO-PANi:CSA fibers increases to an optimal concentration of filler PANi (as shown in Figure 7f for each curve with its molar mass) because the interactions of polymeric molecules (PEO matrix) with the surface of the filler particles lead to restricted mobility of the attached molecules resulting in an increase in elasticity due to longer retardation times.

The elasticity of conductive electrospun fibers (PEO-PANi:CSA fibers) is also strongly affected by its molar mass of host polymer/matrix (PEO). The trending curves of PEO-PANi:CSA fibers (Figure 7f) show that the fibers with the lower molar masses have higher *E* values than higher molar masses of PEO. The reason is that the lower molar mass produces thinner fibers, which are more stretchable and oriented in the electrospinning process due to the lower viscosity. Thus stretching causes high chain orientation along the fiber direction [63,64], which is responsible for higher linear elastic regime (stiffness) in force-strain curves, which is directly dependent on *E*.

The post-annealing process may cause some liberation of residual solvents and reaction between fillers, dopants, and polymer matrices, which could liberate molecules (water/solvent) as by-products (as shown schematically in Figure 5f). The loss of solvent reduces the weight of fibers, which are directly dependent on Young’s modulus. Moreover, the residual solvents act as a plasticizer, which is responsible for low stiffness of un-annealed nanofibers. The removal of plasticizers (solvents) during annealing also increases the degree of crystallinity, which ultimately leads to higher elasticity in conductive fibers [51]. However, Figure 8d shows that Young’s modulus *E* of PEO-PANi:CSA fibers increases as long as the annealing temperature does not deteriorate the polymer. 

**Figure 9:**
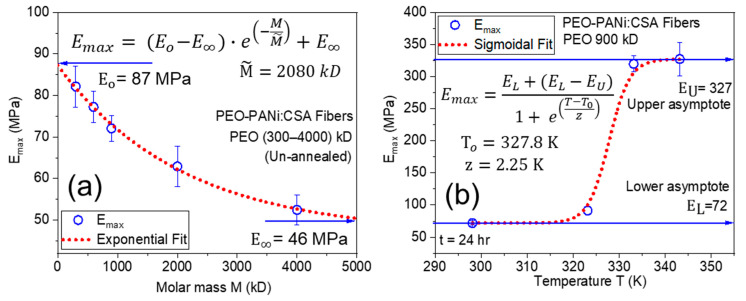
Panel (**a**) shows a negative exponential correlation between maximum Young’s modulus (*E_max_*) and molar mass (*M*) with E_o_ = 87 MPa (at M → 0), E_∞_ = 46 Mpa (at M → ∞) and characteristic molar mass $\tilde{M}$. Panel (**b**) shows the sigmoidal fit function between *E_max_* and temperature *T* with lower (E_L_ = 72 MPa) and upper (E_U_ = 327 MPa) asymptotes, while *T_o_* is the characteristic value of temperature and z describes the slope of fit function.

The behavior of maximum Young’s modulus *E_max_* with molar mass *M* and annealing temperature *T* are shown in Figure 9a,b, respectively. Figure 9a shows that *E_max_* has a negative exponential correlation with molar mass *M* of matrix PEO for PEO-PANi:CSA fibers. *E_max_* tends to E_o_ = 87 MPa for M → 0 and E_∞_ = 46 MPa for M → ∞, respectively. Figure 9b shows the correlation between *E_max_* and temperature *T*. *E_max_* demonstrates nearly sigmoidal behavior, which ends in a plateau as long as the annealing temperature does not deteriorate the polymer. Moreover, the removal of molecules may lead to some physical/chemical bonding among fillers and matrices for improving the conductivity and dimensional stability.

### 2.4. Comparative Study for Applications

**Figure 10:**
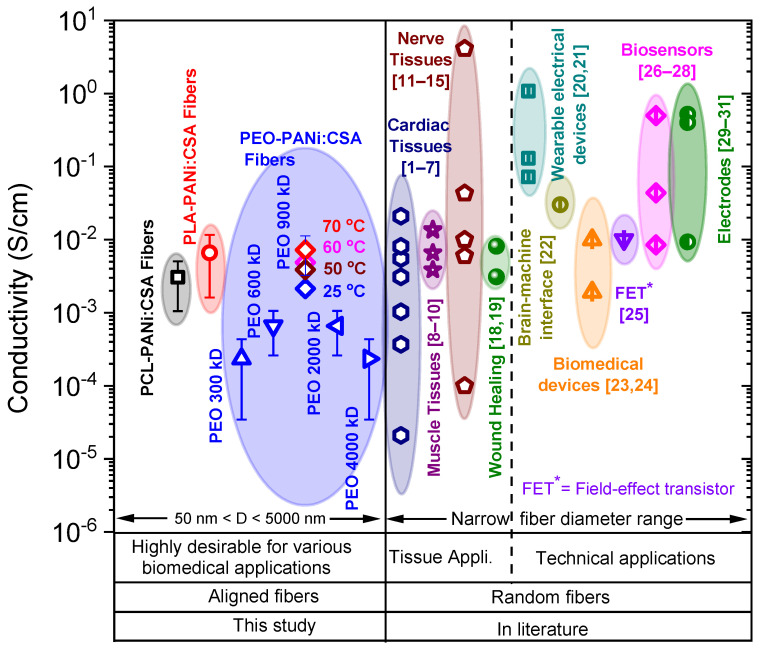
The comparison of conductivities of conductive fibers in this study (PEO-PANi:CSA fibers, PCL-PANi:CSA fibers, and PLA-PANi:CSA fibers) and stated in the literature for various tissue and technical applications. The PEO-PANi:CSA fibers contain conductive fibers for different molar masses of PEO (300–4000) kD. The PEO (900 kD)-PANi:CSA fibers were annealed at 50, 60, and 70 °C for 24 h.

**Figure 11:**
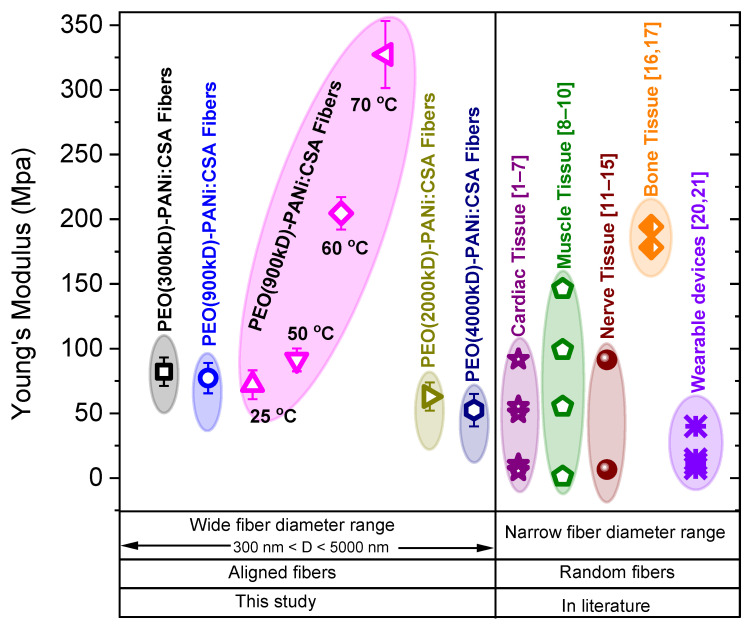
The comparison of Young’s moduli of conductive fibers in this study (PEO-PANi:CSA fibers with 300 kD–4000 kD molar mass of PEO) and stated in the literature for various biomedical applications. The PEO (900 kD)-PANi:CSA fibers were annealed at 50, 60, and 70 °C for 24 h.

The comparative studies are shown in Figure 10 and Figure 11, comparing the conductivities and Young’s moduli (elasticity) of our fabricated ECFCs with other conductive fibers that have been reported on in the literature for various biomedical applications, such as cardiac, nerve, muscle tissues, wound healing, etc., and technical applications, such as biosensors, biomedical devices, electrodes, field-effect transistor, brain-machine interface, and wearable electrical devices. It is significant that the electrical conductivity and mechanical strength (Young’s modulus/stiffness) of our fabricated ECFCs are suitable for a wide range of biomedical and technical applications. Note that our fibers are highly oriented (aligned), while other fibers described in the literature are randomly oriented. Moreover, the fiber-diameter range is a basic requirement for the appropriate usage of fibers in various applications. Our fabricated conductive fibers have a widely variable fiber-diameter (*D*) range from a few tens of nanometers to several micrometers (50 < *D* < 5000 nm). The combination of high orientation, tunable diameter, tunable conductivity, tunable elasticity, and biodegradability makes our nanofibers superior to the fibers described to date and highly desirable for various biomedical and technical applications.

## 3. Materials and Methods

### 3.1. Materials

PEO and PCL purchased from Merck (Sigma-Aldrich, Baden-Württemberg, Germany) and PLA containing 2% D-lactic acid and 98% L-lactic acid (Nature Works, Minnetonka, MN, USA) were used. The order of molar mass for PEO, PCL, and PLA is PEO > PCL > PLA (values are provided under Table 1) and these high molar mass biopolymers were used as polymer matrix. The electrical conductivity of pure PEO, PCL, and PLA fibers was measured and used as conductivity of pure polymer matrix (*σ_m_*) in this study (values are shown in Table 1). The ICPs, such as polyaniline emeraldine base (PANi-EB) doped with (+)-Camphor-10-sulfonic acid (CSA), abbreviated to (PANi:CSA), was used as filler and purchased from Merck (Sigma-Aldrich). PANi was doped with CSA in equal weight ratio (PANi:CSA = 1:1), and the electrical conductivity of doped PANi filler is equal to *σ_f_*, and its value is stated in Table 1. The solvents ethanol (Et-OH) denatured ≥ 99.8, N,N-dimethyl formamide (DMF) ≥ 99.8, and chloroform (CF) were purchased from Merck (Sigma-Aldrich) Germany. The characteristic properties of the used materials, such as weight average molar mass (*Mw*), values of melting point (*mp*), boiling point (*bp*), density (*ρ*), and conductivity (*σ*), are shown in Table 1.

### 3.2. Preparation of Spinning Solutions

**Figure 12:**
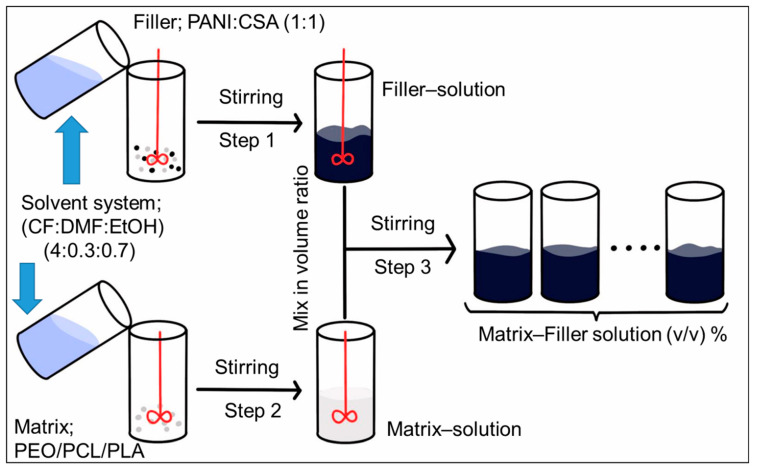
Schematic representation of the procedure for the preparation of spinning solutions.

A schematic for the procedure for preparation of spinning solutions is shown in Figure 12. The polymeric matrix solution and filler solution/dispersion with their respective solvent systems were prepared separately in weight/weight percentage (*w*/*w* %) via step 1 and step 2, respectively. The independently prepared matrix solution and filler solution were mixed in different volume ratios (step 3). The procedure for conversion of (*w*/*w* %) to *v*/*v* % (volume/volume percentage) and derivation of equations for calculating the volume fraction of filler (*φ*) and density (*ρ_c_*) of dried electrospun conductive fiber composites (ECFCs) are explained in detail under Supplementary materials in Section S1.

### 3.3. Electrospinning Set-Up

**Figure 13:**
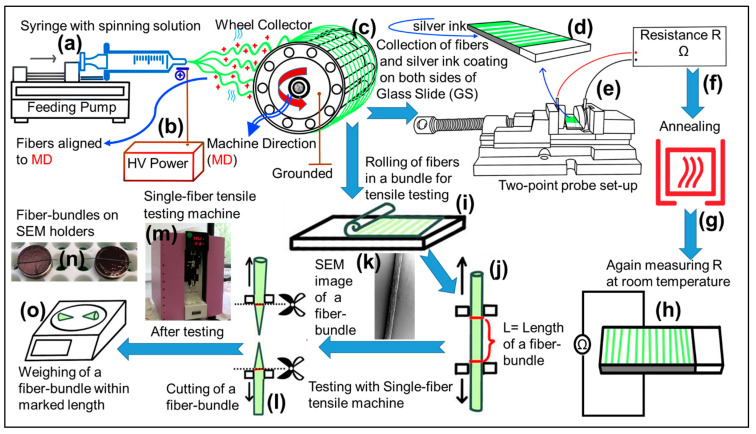
Schematic of electrospinning set-up and procedure for measurement of conductivity and mechanical strength of electrospun conductive fibers. Panels (**a**–**c**) show the feeding pump, high-voltage (HV) power supply, and rotating wheel collector, respectively. The wheel covered with electrospun fibers is aligned to machine direction (MD). Panels (**d**–**f**) show the glass slide (GS), two-point probe set-up, and Ohmmeter for resistance R measurement, respectively. Panels (**g**,**h**) show annealing process and resistance R measurement of annealed fibers, respectively. Panels (**i**–**l**) show the rolling of fibers into a bundle, clamping of a fiber bundle (within red marks), SEM image of a fiber bundle, and testing of a fiber bundle, respectively, using single-fiber tensile testing machine (**m**). Panels (**n**,**o**) show fiber bundles on SEM holders and weighing of tested sample within marked length, respectively.

The electrospinning set-up (schematic shown in Figure 13) consisted of three main parts: a syringe with feeding pump delivering the spinning solution, a high-voltage (HV) power supply (0–60 kV), and a rotating collector for the fibers, which was purpose-built at the University of Erlangen–Nuremberg. A 3D model and its geometrical diagram of the rotating collector are shown in Supplementary materials in Section S3, respectively. The rotating collector consists of two circular terminal plates equipped with circular notches. The notches enable mounting of equally spaced horizontal bars. The bars are held in place by screws. In this study, sixteen bars were used. The spacing between two adjacent bars is 25 mm. The rotating collector has a diameter of 21.2 cm. It can be used up to a speed of 1000 rpm, which corresponds to a tangential velocity of approximately 11 m/s. The matrix filler solutions were poured into 10-mL glass syringe with a blunt 20-gauge stainless steel nozzles with an inner diameter of approximately 0.61 mm and flat tip. The high-voltage power supply (60 kV, DC Linari Engineering S.r.l., and Valpiana, Italy) was used to produce a direct current with the positive polarity on the metallic nozzle, while the collector was grounded. A feeding pump (Linari Engineering S.r.l., and Valpiana, Italy) was used to provide a constant flow rate of the spinning solution. The processing parameters were varied from case to case, and their ranges are: voltage (5–40 kV), flow rate (0.1–4 mL/h), tip-collector distance (5–25 cm), and collector rotation (10–700 rpm). At the optimized processing parameters, the viscoelastic spinning solution was ejected from the spinneret and stretched in the form of a charged jet towards the grounded collector. During the jet’s flight, stretching occurred due to electrical forces, while viscous forces counteracted as discussed in [53] theoretically and in [54] experimentally. The displacement of jets from spinneret to collector and the evaporation (almost complete) of solvent led to solid electrospun conductive fiber composites (ECFCs) on the rotating wheel electrode. These solid ECFCs were collected and transferred onto a glass slide (GS) for further study. A detailed electrospinning theory can be found in the works of Schubert [53,54]

### 3.4. Electrical Conductivity Measurement

An equation was derived to calculate the conductivity (*σ*) of ECFCs. The highly aligned fibers were collected on microscopic glass slides (GS) for the measurement of resistance *R* using an ohmmeter (Keithley 2400). Two opposite sides of GS were coated with silver-ink paste to ensure contact with the electrodes. Complete drying of the paste formed a solid coating layer, as shown in schematic Figure 13.

**Figure 14:**
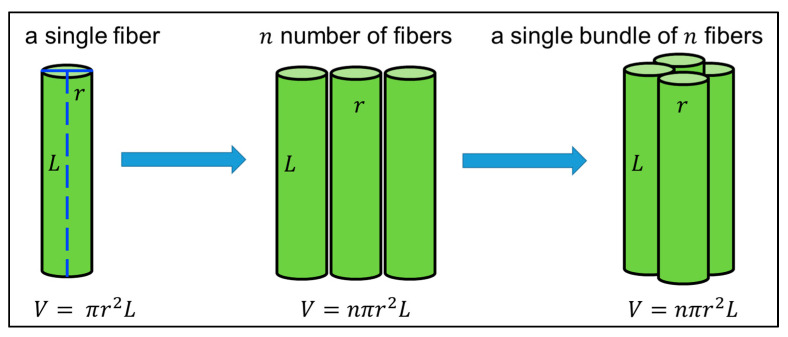
The schematic of a single fiber (length *L* and radius *r*) with *n* number of fibers and a single bundle of *n* fibers for derivation of equations of electrical conductivity (*σ*) and elastic Young’s modulus (*E*) of ECFCs, respectively.

First, consider a single fiber of length *L* and radius *r*, which has cross-sectional area *A_e_* = *πr^2^* (as shown in schematic Figure 14). The fiber has uniform radius along its whole length.

At constant temperature, the resistance of a single fiber *R_e_* were calculated using Equation (3):

(3) $$R_{e}=\frac{\mu_{e}\cdot \;L}{\pi r^{2}}$$

where *μ_e_* is resistivity of a single fiber. For *n* fibers on GS *R* were calculated using Equation (4):

(4) $$R=\frac{\mu \cdot \;L}{n\pi r^{2}}$$

where *R* and *μ* are total resistance and resistivity of *n* fibers, respectively.

As the volume of *n* cylindrical fibers on GS (*V* = *nπr^2^L*) were calculated using Equation (5):

(5) $$n\pi r^{2}L=\frac{W_{c}}{\rho_{c}}$$

where *W_c_* and *ρ_c_* are the final weight and density of dried ECFCs.

Combining and re-arranging Equations (4) and (5) were represented in Equation (6):

(6) $$R=\frac{\mu \cdot \;L^{2}\cdot \;\rho_{c}}{W_{c}}$$

Since conductivity *σ* is the reciprocal of the resistivity *μ* were calculated using Equation (7):

(7) $$\sigma =\frac{1}{\mu }=\frac{\;L^{2}\cdot \;\rho_{c}}{R\;\cdot W_{c}}$$

Using Equation (7), the electrical conductivity *σ* of ECFCs can be calculated using the value of *R*, which is measured using a two-point probe method for a constant voltage (1 V) at room temperature, *W_c_* measured before applying the silver-ink paste, *L* as length of ECFCs (equal to the length of a single fiber), and *ρ_c_* (density of dried ECFCs) calculated from Equation (S14), which is derived and stated under Supplementary materials in Section S1.

### 3.5. Mechanical Strength Measurement

The single-fiber tensile test was performed to determine the mechanical properties of ECFCs. The schematic of the mechanical testing process and photo of the single-fiber tensile testing machine (Vibrodyn 400, Lenzing instruments GmbH & Co. KG, Gampern, Austria) are shown in Figure 13. Under the conditions of 200-mg load and 10-mm clamping length within the VPN program, the fiber bundle was stretched at a speed of 10 mm/min until it broke, providing its force-strain curve. Three replicates were carried out for each kind of fiber. Before the tensile testing, the tensile positions were marked on each fiber bundle. After testing, the fiber bundle was cut off along the marks. The weight of each fiber bundle (within marked length) was measured.

An equation was derived to calculate the elastic Young’s modulus (*E*) of ECFCs. The highly aligned fibers were collected on the glass slide (GS) and rolled to a bundle of fibers (as shown in schematic Figure 14). The SEM images of a fiber bundle with different magnifications have stated under Supplementary materials in Section S4.

Considering a bundle of *n* fibers with length *L* and radius *r*, which has cross-sectional area *A* = *nπr^2^*:

Under optimal conditions, the Young’s modulus of a fiber (*E*) were calculated as Equations (8)–(10):

(8) $$E=\frac{\sigma }{\varepsilon }$$

(9) $$\sigma =\frac{F}{A}$$

(10) $$\varepsilon =\frac{\Delta L}{L}$$

where *σ*, *ε*, *F*, *A*, Δ*L*, and *L* are stress, strain, force, cross-sectional area, change in length, and length of sample, respectively.

As volume of a bundle of fibers (*V* = *nπr^2^L*) were calculated as Equation (11) and (12):

(11) $$V=\frac{W_{c}}{\rho_{c}}$$

(12) V=A·L

where *W_c_* and *ρ_c_* are the final weight (within red marks) and density (as calculated using Equation (S14)) of bundle fiber, respectively.

Combining and re-arranging Equations (8)–(12), were shown as Equation (13) and (14):

(13) $$E=\frac{F\cdot L}{\Delta L}\cdot \frac{\rho_{c}\cdot L}{W_{c}}$$

(14) $$\begin{array}{c} E=k\cdot \frac{\rho_{c}\cdot L}{W_{c}}\\ \mathrm{where}\;k=\frac{F\cdot L}{\Delta L} \end{array}$$

where *k* is the slope of the force-strain curve in the linear elastic region.

The Young’s modulus (*E*) of a bundle of fibers can be determined using Equation (14).

## 4. Conclusions

Highly oriented electrospun conductive fibers with tunable diameter are successfully fabricated using a special rotating collector electrode and allow an accurate determination of electrical conductivity and Young’s modulus (*E*). The fiber diameter is tunable with altering filler-matrix interactions and molar mass of host polymer. 

The electrical percolation threshold (*φ_c_*) is determined by fitting the McLachlan GEM equation to the experimental conductivities of conductive electrospun nanofibers. Higher molar mass produces thicker fiber diameter, and thicker fibers require higher *φ_c_* to develop continuous pathways for charge transport. The high specific surface area of electrospun fibers and high affinity of polyaniline (PANi) for water molecules cause the high inter-grain connectivity and electrical mobility for lowering *φ_c_* in stored conductive fibers. During the annealing process, the molecular chains of host polymers become mobile, and PANi particles acquire a certain degree of freedom to reorganize themselves into additional conductive channels for lowering *φ_c_* in conductive fibers. 

The mechanical properties of fibers are influenced by filler volume fraction (*φ*), molar mass, and post-annealing process. The Young’s modulus *E* increases to its maximum (*E_max_*) and then decreases as a function of filler fraction. The elasticity of conductive electrospun fibers is strongly affected by molar mass of host polymer: the lower the molar mass, higher the elasticity. The lower molar mass produces thinner fibers, which are more stretchable and oriented in the electrospinning process due to the lower viscosity. Thus stretching causes high chain orientation along the fiber direction, which is responsible for higher elasticity. The removal of plasticizers (solvents) during the annealing process increases the degree of crystallinity, which ultimately leads to higher elasticity in conductive fibers. The maximum Young’s modulus *E_max_* exhibits a negative exponential correlation with molar mass of host polymer. The correlation between *E_max_* and temperature shows sigmoidal behavior, which ends in a plateau as long as the annealing temperature does not deteriorate the polymer. 

A trade-off is formed between conductivity and mechanical strength because, on increasing the amount of conductive filler, at first both conductivity and elasticity increase, but the elasticity decreases after optimal filler fraction. Moreover, the fiber diameter, conductivity, and mechanical strength of our fabricated conductive fibers are suitable for a wide range of biomedical and technical applications. The utilization of conductive fibers as nano/micro-carriers in batch and continuous bioprocessing will be evaluated.

## Figures and Tables

**Table 1:** Characteristics and properties of materials used.

Materials	Molar Mass (Mw)	Melting Point (mp)	Density (ρ) at 25 °C	Conductivity (σ)
	(kDa)	(°C)	(g cm^−3^)	(S cm^−1^)
PEO	300 600 900 2000 4000	65 65 65 65 65	1.26 1.26 1.21 1.21 1.21	(8.14 ± 0.3) × 10^−8^ (8.14 ± 0.3) × 10^−8^ (8.14 ± 0.3) × 10^−8^ (8.14 ± 0.3) × 10^−8^ (8.14 ± 0.3) × 10^−8^
PCL	121	60	1.145	(9.62 ± 1.4) × 10^−8^
PLA	109	170	1.24	(81.4 ± 0.9) × 10^−7^
PANi-EB	65	>350	1.101	(1.1 ± 0.3) × 10^−9^ (pressed pellet, ASTM F8) PANi:CSA (1:1) ≈ (100 ± 5)
CSA	232.30 Da	200	1.302	-
H_2_O	18.02 Da	-	1.000 at 3.98 °C	≤(0.05) × 10^−6^
CF	119.38 Da	-	1.48	≤(0.02) × 10^−6^
Et-OH	46.07 Da	-	0.816	Non-conductive
DMF	73.09 Da	-	0.944	≤(0.3) × 10^−6^

## Supplementary Materials

The following are available online at https://www.mdpi.com/article/10.3390/ijms221910295/s1: Section S1, Development of procedure and derivation of equations for calculating volume fraction of filler (φ) and density (ρc) of solid electrospun conductive fiber composites (ECFCs); Section S2, Annealing process for conductive fibers; Section S3, 3D-model of rotating wheel collector electrode; Section S4, SEM images of a fiber bundle.
