# Supplementary material for: Revealing Electrical and Mechanical Performances of Highly Oriented Electrospun Conductive Nanofibers of Biopolymers with Tunable Diameter
Source: Int J Mol Sci. 2021 Sep 24;22(19):10295. doi: 10.3390/ijms221910295 (PMC8509057; doi:10.3390/ijms221910295)
Supplement: Supplementary file 1 [file ijms-22-10295-s001.zip › ijms-1344096-supplementary.pdf]

# Supplementary Materials

## Section S1. Development of procedure and derivation of equations for calculating volume fraction of filler ($\varphi$) and density ($\rho_c$) of solid electrospun conductive fiber composites (ECFCs)

The procedure for calculating volume fraction of filler ($\varphi$) and density ($\rho_c$) of ECFCs has developed in this study. The densities of individual matrices, fillers and solvents have stated in Table 1. Since the final solution for electrospinning contained the mixture of filler, solvent and matrix with different proportion of their respective components. So the densities of filler-mixture ($\rho_f$), solvent-mixture ($\rho_s$), matrix-mixture ($\rho_m$), filler-solution ($\rho_{fs}$) and matrix-solution ($\rho_{ms}$) were calculated step by step by a rule of mixture following Equations (S1–S3) [1].

$$\rho_f = \frac{1}{\frac{MF_{f1}}{\rho_{f1}} + \frac{MF_{f2}}{\rho_{f2}}} \quad \text{(S1)}$$

$$\rho_S = \frac{1}{\frac{MF_{S1}}{\rho_{S1}} + \frac{MF_{S2}}{\rho_{S2}}} \quad \text{(S2)}$$

$$\rho_m = \frac{1}{\frac{MF_{m1}}{\rho_{m1}} + \frac{MF_{m2}}{\rho_{m2}}} \quad \text{(S3)}$$

where; $MF$ is mass fraction and the subscripts $f_1$ & $f_2$, $S_1$ & $S_2$ *and* $m_1$ & $m_2$ show the two components of filler-mixture, solvent-mixture and matrix-mixture, respectively.

As filler-solution and matrix-solution with their respective solvent systems were prepared in weight/weight percentage (w/w %) concentrations with Equation (S4 and S5), respectively.

$$\frac{W}{W}\ \% = \frac{M_f}{M_{fs}} \times 100 = W_{fs} \quad \text{(S4)}$$

$$\frac{W}{W}\ \% = \frac{M_m}{M_{ms}} \times 100 = W_{ms} \quad \text{(S5)}$$

where; $M_f$ and $M_m$ are masses of filler and matrix in their respective masses of filler-solution ($M_{fs}$) and matrix-solution ($M_{ms}$), respectively. For purpose of easiness, $W_{fs}$ and $W_{ms}$ considered equivalent terms to overall masses of filler-solution and matrix-solution, respectively.

The densities of filler-solution ($\rho_{fs}$) and matrix-solution ($\rho_{ms}$) were determined by using Equation (S6 and S7), respectively.

$$\rho_{fs} = \frac{1}{\frac{MF_f}{\rho m} + \frac{MF_s}{\rho s}} \quad \text{(S6)}$$

$$\rho_{ms} = \frac{1}{\frac{MF_m}{\rho m} + \frac{MF_s}{\rho s}} \quad \text{(S7)}$$

where; $MF_m$, $MF_f$ and $MF_s$ are mass fractions of matrix, filler and solvent, respectively.

To convert w/w % concentrations to v/v % (volume/volume percentage) concentrations, Equation S4 and S5 were divided with densities of their respective species by using Equation S6 and S7, respectively. The calculation was shown in Equation (S8 and S9).

$$\frac{V}{V}\,\% = \frac{M_f}{M_{fs}} \times \frac{\rho_{fs}}{\rho_f} \times 100 = V_{fs} \quad \text{(S8)}$$

$$\frac{V}{V}\,\% = \frac{M_m}{M_{ms}} \times \frac{\rho_{ms}}{\rho_m} \times 100 = V_{ms} \quad \text{(S9)}$$

where; $V_{ms}$ and $V_{fs}$ are the volumes of matrix-solution and filler-solution, respectively.

To prepare matrix-filler solution for electrospinning, x-amount of volume of filler-solution ($xV_{fs}$) and y-amount of volume of matrix-solution ($yV_{ms}$) mixed as shown in schematic **Figure S1**.

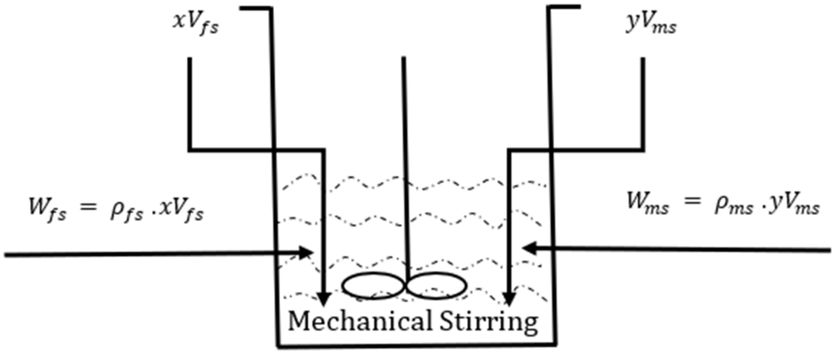


**Figure S1.** Schematic of mixing of x-amount of volume of filler-solution ($xV_{fs}$) and y-amount of volume of matrix-solution ($yV_{ms}$) having densities of $\rho_{fs}$ and $\rho_{ms}$, respectively.

When filler-matrix solution was spun in electrospinning, the solvent got evaporation and solid composite fibers of filler and matrix were obtained, so the volume fraction of filler ($VF_f$ = $\varphi$) in dried solid ECFCs was calculated as Equation (S10);

$$\varphi = VF_f = \frac{xV_{fs}}{xV_{fs}+yV_{ms}} \quad \text{(S10)}$$

The density of solid ECFCs ($\rho_c$) was determined by rule of mixture was calculated as Equation (S11);

$$\rho_c = \frac{1}{\left(\frac{xV_{fs}\cdot\rho_{fs}}{xV_{fs}\cdot\rho_{fs}+yV_{ms}\cdot\rho_{ms}}\right)\cdot\frac{1}{\rho_f}+\left(\frac{yV_{ms}\cdot\rho_{ms}}{xV_{fs}\cdot\rho_{fs}+yV_{ms}\cdot\rho_{ms}}\right)\cdot\frac{1}{\rho_m}} \quad \text{(S11)}$$

To simplify the Equation S11;

$$WF_f = \frac{xV_{fs}\cdot\rho_{fs}}{xV_{fs}\cdot\rho_{fs}+yV_{ms}\cdot\rho_{ms}} \quad \text{(S12)}$$

$$WF_m = \frac{yV_{ms}\cdot\rho_{ms}}{xV_{fs}\cdot\rho_{fs}+yV_{ms}\cdot\rho_{ms}} \quad \text{(S13)}$$

where; $WF_f$ and $WF_m$ are the weight fractions of filler and matrix and calculated by Equation S12 and S13, respectively. By using Equation S11, S12 and S13, The density of solid ECFCs ($\rho_c$) was calculated by a simplified form of rule of mixture (Equation S14);

$$\rho_c = \frac{1}{\frac{WF_f}{\rho_f}+\frac{WF_m}{\rho_m}} \quad \text{(S14)}$$

**Section S2. Annealing process for conductive fibers**

All samples of conductive fibers (ECFCs) annealed isothermally in vacuum oven at their respective temperatures for 24 hours. All ECFCs samples collected on glass slides. First, the vacuum oven was turned on and heated upto required value of temperature and after reaching to stable heating conditions, the samples were put inside to airtight oven and turned on the vacuum to eliminate all residual vapours during heating. After every required annealing temperature and time, the sample were taken out from oven to allow them cooling at room temperature of laboratory. In whole study, the samples for each category annealed separately to avoid from contamination.

## Section S3. 3D-model of rotating wheel collector electrode

A 3D model and its geometrical diagram are shown in Figure S2 and S3, respectively

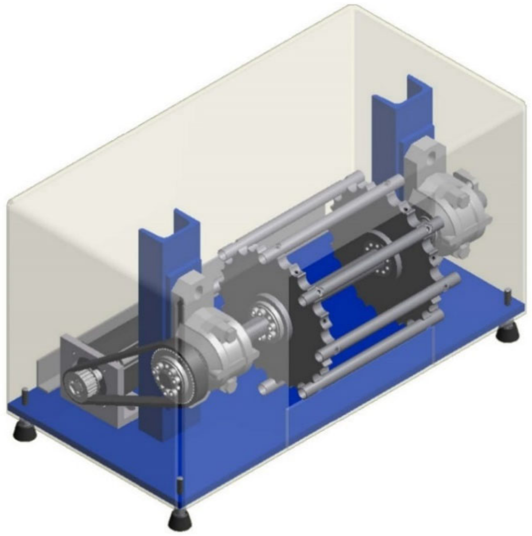

**Figure S2.** Custom made 3D-model of rotating wheel collector electrode, which is driven by a v-belt [2].

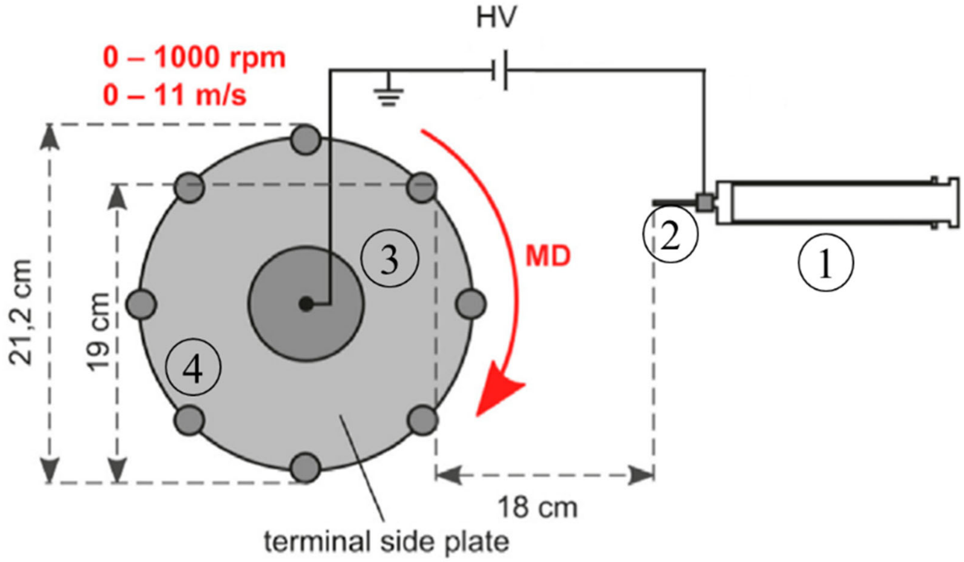


**Figure S3.** Geometrical diagram of rotating wheel collector electrode where; (1) Syringe with solution; (2) Needle (Anode); (3) Rotating collector (Cathode); (4) Metallic circular bars for collecting fibers.

## Section S4. SEM images of a fiber bundle

The SEM images of a fiber-bundle with different magnifications.

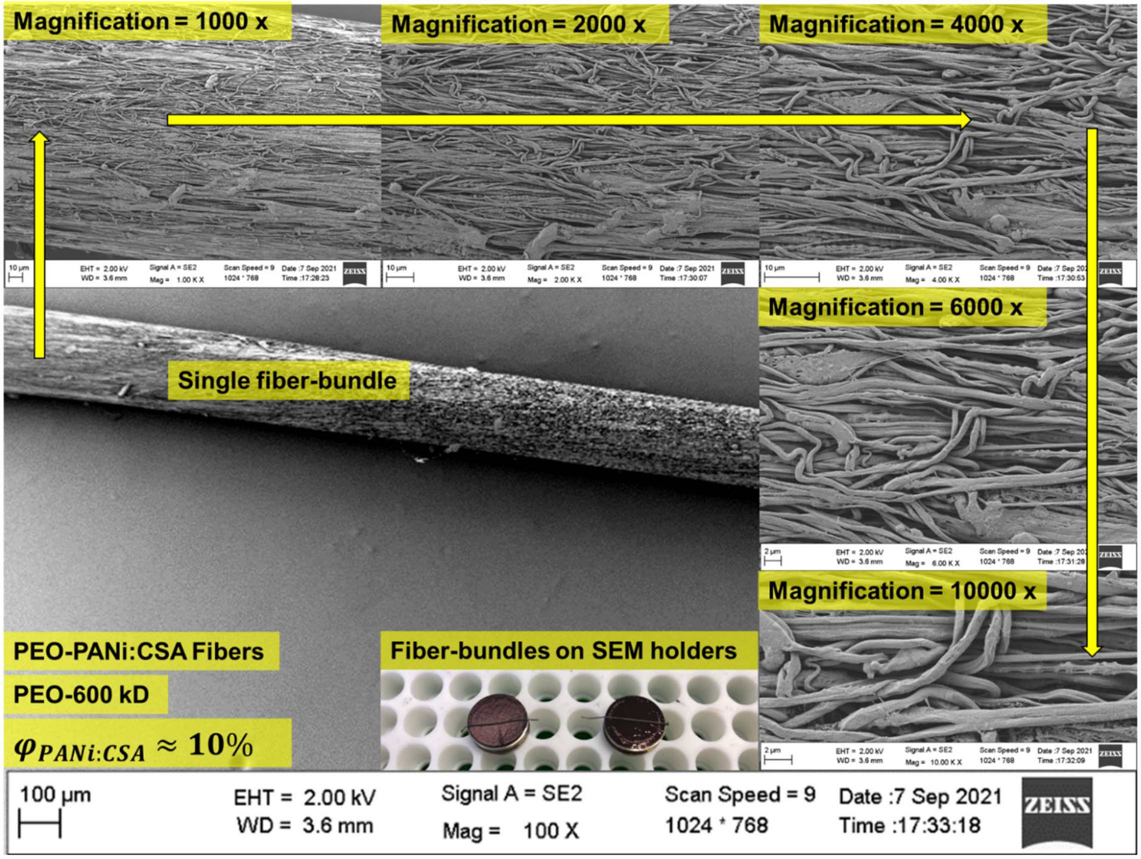


**Figure S4.** SEM images of a fiber bundle

## References

1. Tam, D.; Ruan, S.; Gao, P.; Yu, T. 10 - High-performance ballistic protection using polymer nanocomposites. In *Advances in Military Textiles and Personal Equipment: Woodhead Publishing Series in Textiles*; Sparks, E., Ed.; Woodhead Publishing, **2012**; pp 213–237. DOI: 10.1533/9780857095572.2.213.
2. Doergens, A.; Roether, J. A.; Dippold, D.; Boccaccini, A. R.; Schubert, D. W. Identifying key processing parameters for the electrospinning of aligned polymer nanofibers. *Materials Letters* **2015**, *140*, 99–102. DOI: 10.1016/j.matlet.2014.10.150.
